# *Clostridium difficile* in Retail Meat Products, USA, 2007

**DOI:** 10.3201/eid1505.081071

**Published:** 2009-05

**Authors:** J. Glenn Songer, Hien T. Trinh, George E. Killgore, Angela D. Thompson, L. Clifford McDonald, Brandi M. Limbago

**Affiliations:** University of Arizona, Tucson, Arizona, USA (J.G. Songer, H.T. Trinh); Centers for Disease Control and Prevention, Atlanta, Georgia, USA (G.E. Killgore, A.D. Thompson, L.C. McDonald, B.M. Limbago)

**Keywords:** *Clostridium difficile* and associated disease, enteric diseases, retail meats, food animals, dispatch

## Abstract

To determine the presence of *Clostridium difficile*, we sampled cooked and uncooked meat products sold in Tucson, Arizona. Forty-two percent contained toxigenic *C. difficile* strains (either ribotype 078/toxinotype V [73%] or 027/toxinotype III [NAP1 or NAP1-related; 27%]). These findings indicate that food products may play a role in interspecies *C. difficile* transmission.

The incidence and severity of *Clostridium difficile* infections (CDIs) are increasing in North America (*1*), probably because of emergence of an epidemic strain (NAP1/BI/027, toxinotype [TT] III) (*2*,*3*). *C. difficile* transmission occurs primarily in healthcare facilities, but community-associated CDI (CA-CDI) appears to be increasing and may now account for 20%–45% of positive diagnostic assay results (*4*,*5*). Up to 35% of patients with CA-CDI report no antimicrobial agent use within 3 months before disease onset (*4*,*5*), although nonantimicrobial drugs (e.g., proton pump inhibitors, nonsteroidal antiinflammatory agents) are also implicated as risk factors (*4*). Sources of *C. difficile* acquisition in community settings are unknown.

CDI is increasingly important in food animals (*6*). Infection rates of >95% have been documented among neonatal pigs in farrowing facilities, resulting in diarrhea and typhlocolitis (*6*). Toxigenic *C. difficile* is also implicated as a cause of diarrhea in calves (*7*). *C. difficile* was identified in raw meat intended for pet consumption (*8*) and in ≈20% of retail ground beef in Canada (*9*). We report the isolation of *C. difficile* from uncooked and ready-to-eat meats in retail markets in a US metropolitan area.

## The Study

Packaged meats were purchased from 3 national-chain grocery stores in the Tucson, Arizona, area on 3 occasions at 1-month intervals from January to April 2007. Products sampled were both uncooked (ground beef, ground pork, ground turkey, pork sausage, and pork chorizo) and ready to eat (beef summer sausage, pork braunschweiger) (Table). Pork chorizo was produced and distributed locally; all other samples were national brands. Products with different sell-by dates (a surrogate for production date) were sampled for each meat type. Samples were not representative of all meat products in each grocery store.

**Table:** Source and characteristics of *Clostridium difficile* isolates obtained from retail meats sold in Tuscon, Arizona, USA, 2007*

Meat product	No. samples cultured	Total no. (%) positive	Ribotype	Toxinotype	Δ*tcdC*, bp†	PFGE type	No. (%) positive
Ground beef (uncooked)	26	13 (50)	027	III	18	NAP1	1 (3.8)
						NAP1-related	2 (7.7)
			078	V	39	NAP7	8 (30.8)
						NAP8	2 (7.7)
Summer sausage (ready to eat)	7	1 (14.3)	027	III	18	NAP1	1 (14.3)
Ground pork (uncooked)	7	3 (42.9)	027	III	18	NAP1-related	1 (14.3)
			078	V	39	NAP7	2 (28.6)
Braunschweiger (ready to eat)	16	10 (62.5)	027	III	18	NAP1	2 (12.5)
						NAP1-related	1 (6.2)
			078	V	39	NAP7	7 (43.8)
Chorizo (uncooked)	10	3 (30.0)	027	III	18	NAP1-related	1 (10.0)
			078	V	39	NAP7	2 (20.0)
Pork sausage (uncooked)	13	3 (23.1)	027	III	18	NAP1-related	1 (7.7)
			078	V	39	NAP7	2 (15.4)
Ground turkey (uncooked)	9	4 (44.4)	078	V	39	NAP7	4 (44.4)
Totals	88	37 (42.0)	027	III	18	NAP1	4 (4.4)
						NAP1-related	6 (6.7)
			078	V	39	NAP7	25 (27.8)
						NAP8	2 (2.2)

*All samples were positive for *cdtB*, which encodes the binding component of binary toxin. PFGE, pulsed-field gel electrophoresis. †Deletions in *tcdC* regulatory gene.

For each sample, 1 g of meat was added to two 10-mL tubes of prereduced brain heart infusion (BD, Franklin Lakes, NJ, USA), which had been supplemented with 0.5% yeast extract (BD), 0.05% DL-cysteine (Sigma-Aldrich, St. Louis, MO, USA), and 0.1% taurocholate (MP Biomedicals, Solon, OH, USA). One tube was heat shocked (80°C, 10 min), and both were then incubated anaerobically at 37°C for 72 h. Aliquots were subcultured onto taurocholate cycloserine cefoxitin fructose agar (TCCFA) (*10*) and incubated anaerobically for 24–72 h at 37°C. Colonies were subcultured onto anaerobic blood agar, TCCFA (with or without antimicrobial agents), and confirmed as *C. difficile* by *p*-cresol odor, yellow-green fluorescence under UV illumination, a positive L-proline aminopeptidase reaction, and negative indole reaction.

Isolates were characterized by PCR ribotyping (*11*), toxinotyping (*3*), and pulsed-field gel electrophoresis (PFGE) (*12*). Presence of *tcdA, tcdB, cdtB* (binary toxin), and deletions in *tcdC* was determined by PCR (*2*).

MICs were determined by Etest (AB Biodisk, Solna, Sweden) on *Brucella* blood agar with vitamin K and hemin (Remel, Lenexa, KS, USA) that was incubated anaerobically at 35°C. Reference interpretive criteria for *C. difficile* susceptibility to clindamycin and moxifloxacin were used; MICs for levofloxacin and gatifloxacin were interpreted by using criteria for moxifloxacin (*13*). *Bacteroides fragilis* ATCC 25285, *B. thetaiotaomicron* ATCC 29741, *C. difficile* ATCC 700057, and *Enterococcus faecalis* ATCC 29212 were included as controls.

Proportions were compared by χ^2^ or Fisher exact test. Thirty-seven (42.0%) of 88 retail meats yielded *C. difficile*, including 42.4% of beef, 41.3% of pork, and 44.4% of turkey products (Table). Ready-to-eat products were more commonly culture positive (11/23; 47.8%) than were uncooked meats (26/65; 40.0%), although the difference was not significant (p = 0.34). The highest percentages of *C. difficile* isolates were recovered from pork braunschweiger (62.5%) and ground beef (50.0%). Culture-positive results came from both heat-shocked and non–heat-shocked cultures, whereas culture-negative specimens were negative in both types of culture, and no specimen was positive by both methods (not shown). No association was found with the meat processor, the sell-by date, the store, or the month sampled (not shown). Multiple independent cultures from 2 braunschweiger samples yielded indistinguishable isolates in the same meat sample (10/10 from 1 package and 12/12 from another; not shown), which suggests that a single strain may predominate when *C. difficile* is present. Our percentage of recovery of *C. difficile* from retail meat products is higher than that reported (20%) in a similar study of Canadian ground beef (*9*), possibly because of differences in culture methods, the meats sampled, or national or geographic variation.

Isolates were grouped into ribotype 078/TT V (27/37, 73.0%) and ribotype 027/TT III (10/37, 27.0%). Strain types were not specific to meat type, store, or sampling month (Table). All isolates were PCR positive for binary toxin (*cdtB*), *tcdA,* and *tcdB*. Characteristic 18-bp and 39-bp deletions in *tcdC* were present in 027/TT III and 078/TT V isolates, respectively (*2*,*12*). PFGE divided 027/ TT III isolates into NAP1 (>80% related to human NAP1) and NAP1-related (78% related to human NAP1) groups and 078/TT V isolates into NAP7 and NAP8 groups (Figure).

**Figure:**
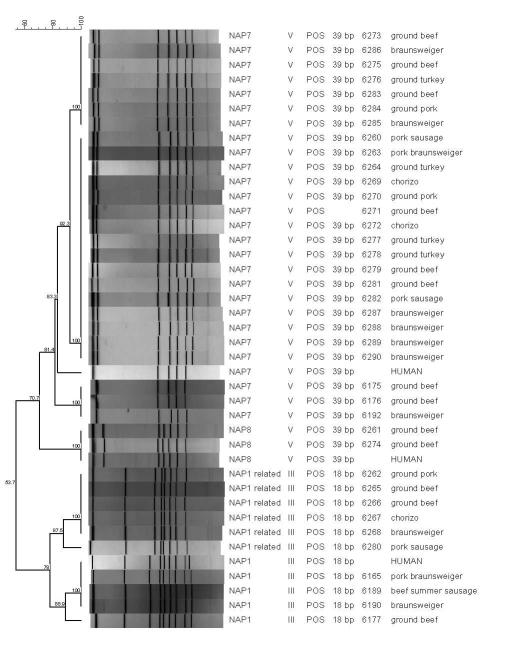
Origin, NAP types, and relatedness of strains from foods and humans, Arizona, USA**,** 2007. All strains were positive by PCR for binary toxin. Scale bar indicates genetic relatedness. Tox, toxinotype; Ref, reference; NAP1-r, NAP1-related.

Ribotype 027 isolates are described almost exclusively in context of the current human epidemic strain, NAP1/027/TT III (*2*). In this study, we also found 027/TT III isolates that were only 78% similar to NAP1 (i.e., NAP1-related). Ribotype 078 strains were previously uncommon causes of healthcare-associated CDI in humans (*12*), but now they are emerging in pigs and calves with diarrhea ( *7;* J.S. Weese, pers. comm.) and in persons with CDI (*12*). Two epidemiologically unrelated 078/TT V isolates from human CDI patients are indistinguishable by PFGE from pig isolates (*12*).

The 078/TT V isolates were uniformly susceptible to levofloxacin, moxifloxacin, and gatifloxacin. Like human TT V isolates (*12*), most 078/TT V meat isolates were nonsusceptible to clindamycin (56% resistant, 41% intermediate). This may not be surprising given the widespread use of tylosin, erythromycin, virginiamycin, and lincomycin in food animals and the potential for selection of macrolide-lincosamide-streptogramin resistance (*14*).

NAP1 isolates have demonstrated high-level resistance to levofloxacin, moxifloxacin, gatifloxacin (>32 μg/mL), and clindamycin (>256 μg/mL), consistent with current human strains (*2*). NAP1-related isolates were susceptible to levofloxacin, moxifloxacin, and gatifloxacin but resistant to clindamycin, similar to the pattern of historic NAP1 strains (*2*).

## Conclusions

Fluoroquinolones are widely used in human therapy, and the current epidemic strain may have emerged because of its resistance to these agents. Fluoroquinolone use is limited in food animal production (*14*), with the exception of enrofloxacin for treatment of bovine respiratory disease (now approved for use in swine).

The source of *C. difficile* in retail meats may involve antemortem deposition of spores in the animal’s muscle or other tissues, fecal or environmental contamination of carcasses, or contamination during processing. Spores could persist in packing plants, resulting in contamination of carcasses or food products during processing. Contamination may also occur in retail meat markets.

Direct or indirect human-to-human transmission is responsible for most healthcare-related CDIs (*15*) and most likely contributes to CA-CDI. Therefore, stopping such transmission remains the critical control point for preventing most human CDIs. Nonetheless, our findings highlight the potential both for selection of virulent or resistant strains in animals and interspecies transmission through the food supply. Our data do not prove transmission of *C. difficile* from foods to humans but highlight the need for studies to characterize risks posed by this organism in the human food supply.

## References

[1] Zilberberg MD, Shorr AF, Kollef MH. Increase in adult *Clostridium difficile*–related hospitalizations and case-fatality rate, United States, 2000–2005. Emerg Infect Dis. 2008;14:929–31. 10.3201/eid1406.071447 18507904 PMC2600276

[2] McDonald LC, Killgore GE, Thompson A, Owens RC Jr, Kazakova SV, Sambol SP, An epidemic, toxin gene-variant strain of *Clostridium difficile.* N Engl J Med. 2005;353:2433–41. 10.1056/NEJMoa051590 16322603

[3] Rupnik M, Avesani V, Janc M, von Eichel-Streiber C, Delmée M. A novel toxinotyping scheme and correlation of toxinotypes with serogroups of *Clostridium difficile* isolates. J Clin Microbiol. 1998;36:2240–7. 9665999 10.1128/jcm.36.8.2240-2247.1998 PMC105025

[4] Dial S, Delaney JA, Schneider V, Suissa S. Proton pump inhibitor use and risk of community-acquired *Clostridium difficile*–associated disease defined by prescription for oral vancomycin therapy. CMAJ. 2006;175:745–8. 10.1503/cmaj.060284 17001054 PMC1569908

[5] Kutty PK, Benoit SR, Woods CW, Sena AC, Naggie S, Frederick J, Assessment of *Clostridium difficile*–associated disease surveillance definitions, North Carolina, 2005. Infect Control Hosp Epidemiol. 2008;29:197–202. 10.1086/528813 18241032

[6] Songer JG. The emergence of *Clostridium difficile* as a pathogen of food animals. Anim Health Res Rev. 2004;5:321–6. 10.1079/AHR200492 15984348

[7] Hammitt MC, Bueschel DM, Keel MK, Glock RD, Cuneo P, DeYoung DW, A possible role for *Clostridium difficile* in the etiology of calf enteritis. Vet Microbiol. 2008;127:343–52. 10.1016/j.vetmic.2007.09.002 17964088 PMC7131641

[8] Weese JS, Rousseau J, Arroyo L. Bacteriological evaluation of commercial canine and feline raw diets. Can Vet J. 2005;46:513–6. 16048011 PMC1140397

[9] Rodriguez-Palacios A, Staempfli HR, Duffield T, Weese JS. *Clostridium difficile* in retail ground meat, Canada. Emerg Infect Dis. 2007;13:485–7. 17552108 10.3201/eid1303.060988 PMC2725909

[10] Wilson KH, Kennedy MJ, Fekety FR. Use of sodium taurocholate to enhance spore recovery on a medium selective for *Clostridium difficile.* J Clin Microbiol. 1982;15:443–6. 7076817 10.1128/jcm.15.3.443-446.1982 PMC272115

[11] Stubbs SL, Brazier JS, O'Neill GL, Duerden BI. PCR targeted to the 16S–23S rRNA gene intergenic spacer region of *Clostridium difficile* and construction of a library consisting of 116 different PCR ribotypes. J Clin Microbiol. 1999;37:461–3. 9889244 10.1128/jcm.37.2.461-463.1999 PMC84342

[12] Jhung MA, Thompson AD, Killgore GE, Zukowski WE, Songer G, Warny M, Toxinotype V *Clostridium difficile* in humans and food animals. Emerg Infect Dis. 2008;14:1039–45. 10.3201/eid1407.071641 18598622 PMC2630049

[13] Clinical and Laboratory Standards Institute. Methods for antimicrobial susceptibility testing of anaerobic bacteria; approved standard, 7th ed. CLSI document M11–A7. Wayne (PA): The Institute; 2007. p. 50.

[14] Giguère S. Antimicrobial therapy in veterinary medicine, 4th ed. Ames (IA): Blackwell Publishers; 2006. p. xvi.

[15] Dubberke ER, Reske KA, Yan Y, Olsen MA, McDonald LC, Fraser VJ. *Clostridium difficile*–associated disease in a setting of endemicity: identification of novel risk factors. Clin Infect Dis. 2007;45:1543–9. 10.1086/523582 18190314

